# The Cashew Pseudofruit (*Anacardium occidentale*): Composition, Processing Effects on Bioactive Compounds and Potential Benefits for Human Health

**DOI:** 10.3390/foods13152357

**Published:** 2024-07-26

**Authors:** Carina Gutiérrez-Paz, María-Constanza Rodríguez-Moreno, María-Soledad Hernández-Gómez, Juan Pablo Fernández-Trujillo

**Affiliations:** 1 Instituto de Ciencia y Tecnología de Alimentos (ICTA), Universidad Nacional de Colombia, Carrera 30 Calle 45, Bogotá 111321, Colombia; cgutierrezp@unal.edu.co (C.G.-P.); mshernandez@unal.edu.co (M.-S.H.-G.); 2 Centro de Pensamiento Turístico de Colombia, Escuela de Turismo y Gastronomía, Fundación Universitaria Cafam, Ak 68 #90-88, Bogotá 111211, Colombia; maria.rodriguez@unicafam.edu.co; 3 Department of Agronomical Engineering, Technical University of Cartagena, Paseo Alfonso XIII, 48, ETSIA, 30203 Cartagena, Murcia, Spain

**Keywords:** *Anacardium*, antioxidant, bioactive compounds, diabetes, obesity, dyslipidemias, functional foods, peduncle, polyphenols, by-products

## Abstract

The fruit of the cashew, a tree belonging to the family Anacardiaceae, is composed of approximately 10% nut (cashew) and 90% stalk or pseudofruit, usually discarded in situ and fermented in the soil. This review identifies cashew pseudofruit’s physicochemical characteristics and bioactive compounds and their possible relationship to health benefits. Different processing techniques have been used to preserve the pseudofruit, and the effect of these techniques on its nutrients is also reviewed in this work. Cashew is a highly perishable product with moisture content above 80% w/w and 10% w/w sugars. It also has a high content of polyphenols, flavonoids, and tannins and high antioxidant properties that are best preserved by nonthermal processing techniques. The pseudofruit presents the high inhibitory activity of α-amylase and lipase enzymes, has anti-inflammatory and body weight reduction properties and healing activity, and controls glucose levels, insulinemia, and insulin resistance. For all these reasons, cashews have been promoted as a propitious food/ingredient for preventive and therapeutic management of different pathologies such as diabetes, dyslipidemia, obesity, hypertension, fatty liver, and acne. Moreover, it has positive effects on the intestinal microflora, among others. This pseudofruit has a high potential for the development of functional foods.

## 1. Introduction

The cashew tree (*Anacardium occidentale*) belongs to the family Anacardiaceae under the order Sapindales. The family comprises 60 to 74 genera and 400 to 600 species [1]. The tree is native to Brazil and was introduced by European explorers to Asia and Africa in the 16th century [2]. About 21 species have been identified through primary taxonomy, and more than 70% are species from Brazil. The main diversification of *Anacardium* is found in Amazonia and, secondarily, in Brazil, mainly in northeastern Brazil for cultivated species. The cashew tree is tropical dicotyledonous, composed of a fully developed root consisting of a main root surrounded by an extensive and well-developed network of lateral roots, 90% of which are found in the soil layer at a depth of 15 to 32 cm. The tree has low branches with an average height of 5 to 8 m and 10 to 12 m wide, simple leaves ranging in size from 6 to 24 cm in length and 15 cm wide, and flowers called terminal panicles that are both male (staminate) and bisexual or hermaphrodite in the same panicle.

The fruit of this tree is called nut or cashew (the main product of the crop) and is gray kidney-shaped, wrapped by the shell or testa. Its average weight is 7 to 12 g. The cashew apple corresponds to the peduncle of the fruit and is called pseudofruit, with a fleshy and juicy consistency. Its size is elongated, round, and pear-shaped. Depending on the variety, its texture, color, and flavor differ [3]. The nut represents 8% to 12% of the total weight of the fruit and is the most traded part. The peduncle manifests a behavior like that of a non-climacteric fruit and presents a high respiration rate in postharvest [4], so it should be harvested fully ripe. This peduncle is yellow or bright red, with dimensions of 4 to 12 cm long, which corresponds to 88% to 92% of the fruit [1,5,6]. The separation of the nut from the peduncle produces a rapid degradation of the latter with dehydration, softening, and oxidation of the area where there was peduncle-nut contact.

The global production of cashew nuts has been dynamic, with notable shifts in leadership. In 2016, the production reached 4.89 million tons, with Vietnam emerging as the largest producer [2]. However, the 2019/2020 production saw India taking the lead, producing 4.6 million tons of nuts and contributing 176,600 tons of kernel (nut). Despite these fluctuations, the demand for cashews continues to grow at an annual rate of approximately 7% [7], underscoring the global significance of this crop.

The cashew agroindustry has a well-defined geographic productive occupation. More than half of the world’s production comes from African countries (57%), while Brazil is the leading and most representative producer in the Americas [7]. Regarding Colombia, from 2012 to 2019, 42,562 hectares were planted, resulting in the production of 128,121 ton, and a yield of 3.6 t/ha (nut), with Orinoco being the region with the largest production [8]. The main business for cashews is the commercialization of the kernel for global export, mainly to the United States, Europe, and some Asian cities [2].

Each ton of cashew nut produced yields 10 to 15 tons of cashew apple as a by-product [3]. In almost all countries, apples are left on the ground as waste when the nuts are harvested, resulting in low income for farmers [3,5,9]. The pseudofruit corresponds to 90% of the crop; therefore, the nut only accounts for the remaining 10% [10], and apple losses have been reported to be between 85% and 90% worldwide [3,11]. Postharvest losses of cashew apples can reach 90% due to the high metabolic activity of the fruit [12]. The production of CO_2_ from the decomposition of the highly perishable pseudofruit corresponds, on average, to emissions of 2.1–4.5 kg of CO_2_ per kilogram of product, which also contributes to climate change [5]. To mitigate CO_2_ emissions due to decomposition, different studies seek to increase cashew’s shelf life so that they can be marketed and used to develop products by consumers and the food industry [3].

This review aims to identify the physicochemical characteristics and bioactive compounds of the cashew pseudofruit, their possible relationship to human health benefits, and the effect of different preservation or processing technologies on the fruit’s nutrients. It is complemented by a small initial literature review and data from our own ongoing research of two cashew varieties.

## 2. Cashew Research

**Figure 1:**
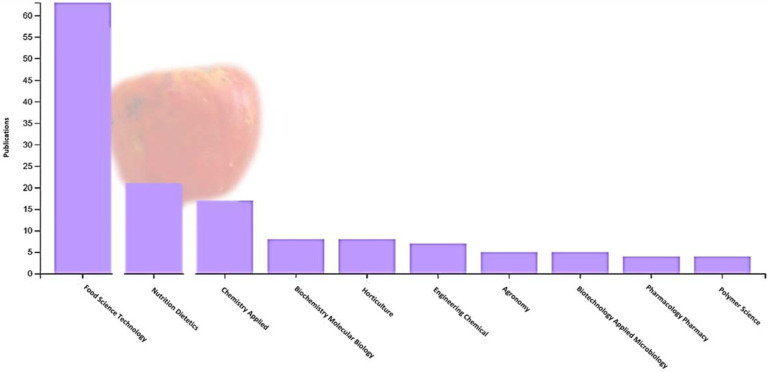
Area of research interest in cashew pseudofruit (*Anacardium occidentale*) according to the Web of Science database (2017–2023).

**Figure 2:**
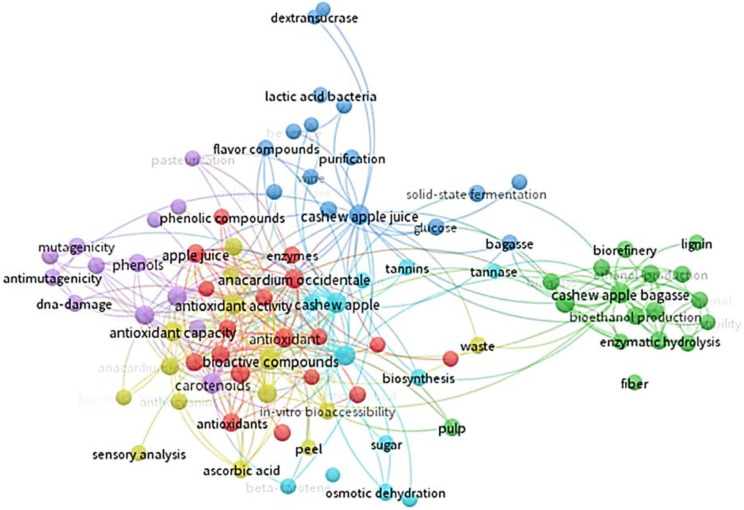
Technological monitoring of the different research conducted on cashew pseudofruit (*Anacardium occidentale*) using the VOSviewer tool 1.6.20 (2008–2023). From Web of Science database (Clarivate Analytics^®^, Philadelphia, PA, USA).

Cashew apples can be preserved for up to 15 to 20 days after harvest using refrigeration at 5 °C and 85% to 90% relative humidity. The fruit is not susceptible to chilling injury, and temperatures of 0 to 1.7 °C could be used for 2 weeks to avoid excessive losses. Packaging cashew apples in modified atmospheres extends storage life for 10 to 25 days, depending on the cultivar [12]. However, due to the high percentage of waste generated by the pseudofruit, research on fruit attributes has been carried out in the last 10 years (particularly during the last 5 years). The countries with the most research are Brazil and India, and the areas of interest focus especially on food science and technology, applied biotechnology, applied chemistry, and nutrition and dietetics (Figure 1). The research conducted focuses on the fruit’s bioactive compounds, antioxidant activity, phenolic compounds, ascorbic acid content, and carotenoids (Figure 2).

**Figure 3:**
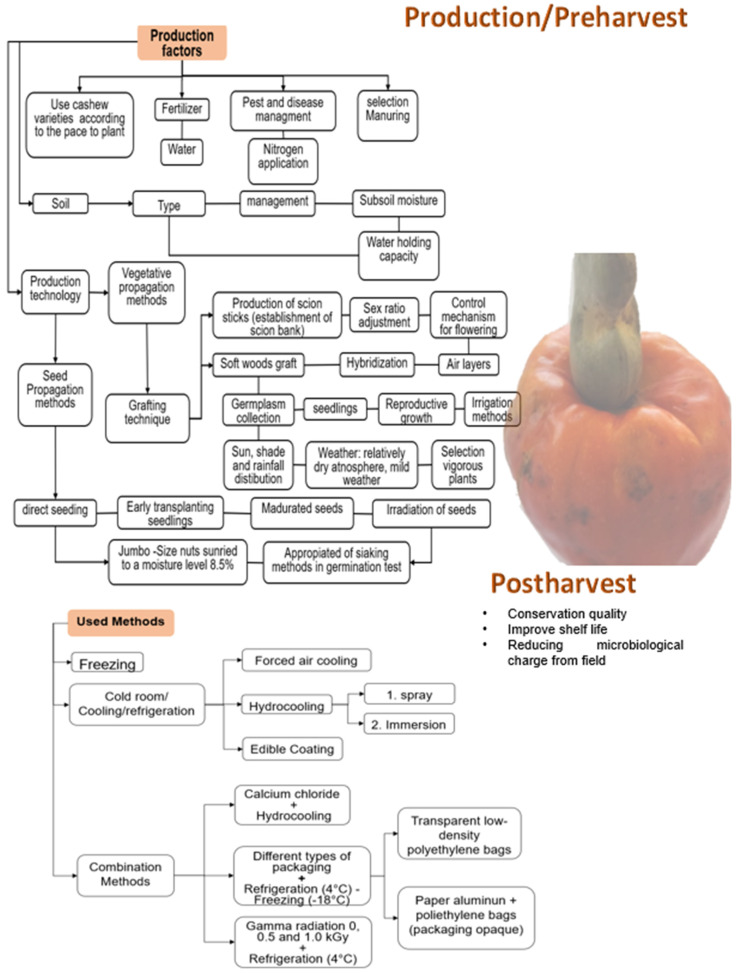
Techniques developed through specific literature and used to improve cashew (*Anacardium occidentale*) production and preserve the pseudofruit freshness after harvest [4,13,14,15,16,17,18].

The rapid loss of quality of fresh fruit is due to the high respiratory rate, changes in the cell wall composition that generate cellular content leaks, and the integrity of the plasma membrane, which affect the firmness of the fruit. Moreover, there is weight loss due to water loss, decreased ascorbic acid rate, and lipid membrane peroxidation [4,12]. For all these reasons, studies have been carried out to improve the shelf life and preserve the fruit’s quality using different techniques that prove the delay of deterioration both in production (pre-harvest) and postharvest (Figure 3).

## 3. Physicochemical Characteristics

**Figure 4:**
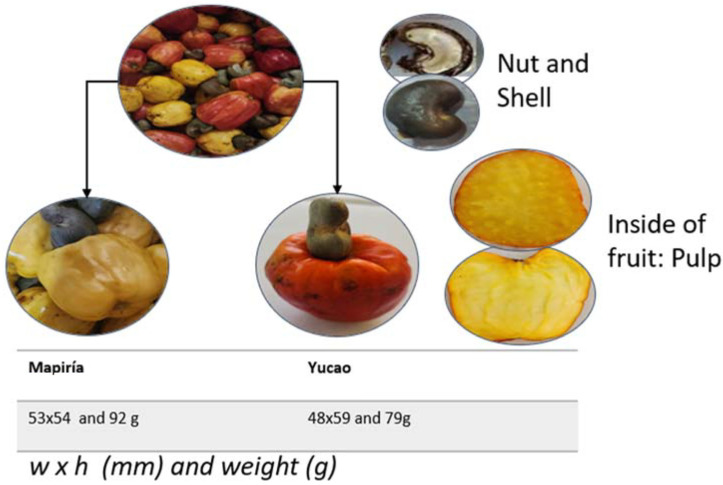
A mixture of fruits of the Yucao and Mapiría cashew pseudofruit (*Anacardium occidentale*) varieties from Puerto Carreño, Vichada, Colombia (top); details of the fruit on the right; and details of both varieties, dimensions, and average weight at the bottom (own data).

Some data from our own research was obtained following protocols established for the traits measured [19,20,21]. The pseudofruit’s average weight is 80 to 160 g, and its length is 60 to 100 mm (Figure 4). Although the pseudofruit of the varieties analyzed falls within the ranges recorded by [21], most other studies do not, so it is impossible to compare with other studies.

**Figure 5:**
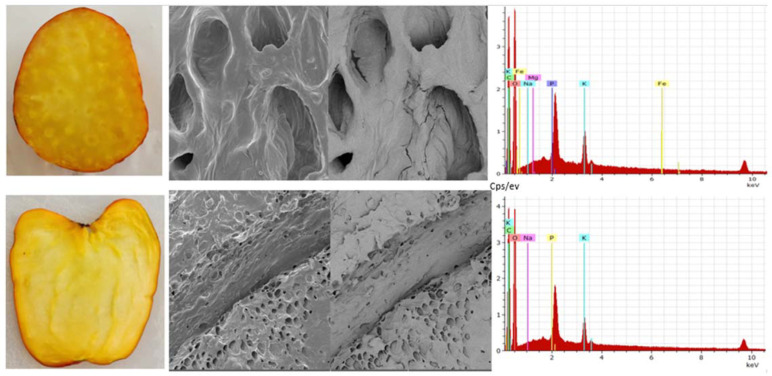
Cashew apple (*Anacardium occidentale*), Yucao variety. Transversal and longitudinal section (**left**), observation by scanning electron microscopy (TESCAN-VEGA 3, Vortex Company, Bogotá, Colombia) (**center**) at upper image field of view 100 μm, SEM HV 10.0 kV, SEM MAG 1.00 kx, WD 15.00 mm and lower image field of view 500 μm, SEM HV 10.0 kV, SEM MAG 200×, WD 14) and composition (**right**) (own data).

The cashew pseudofruit has an average porosity of 46% at harvest [21]. It is ellipsoidal, with colors varying between reddish and yellowish (Figure 4). It exhibits low thermal conductivity, which may be related to its ultrastructure (Figure 5), so thermal processing of the fruit requires a lot of energy. However, these values and the texture depend on the variety, ripening stage, and growing region.

The pseudofruit is harvested considering the kernel’s conditions and not the pseudofruit’s characteristics. For example, when evaluating the penetration force (bioyield point) with a TXT-TA2 texturometer (Stable micro Systems, Bogotá, Colombia) and a 4.6 mm, punch on two varieties 72 h after harvesting (plus 3 more hours after separating the kernel), a mean ± SD was found (*n* = 10) value of 226.1 g ± 63.2 for the Yucao variety and 284.4 g ± 82.9 for the Mapiría variety.

On the inside, the fruit presents particular characteristics that can be analyzed on longitudinal and transversal sections, allowing for the observation of the pseudofruit’s morphology and composition (Figure 5).

In the cross-section of the pseudofruit, there are sections of specialized xylem cells as vessels (vessel elements) along the pseudofruit where some nutrients flow from the apple to the nut. When zooming in on one of these, their general composition was evaluated, and it was found that oxygen and carbon predominate, followed by potassium, sodium, and phosphorus in smaller quantities. Subsequently, when evaluating the vessels or tracheids identified in the longitudinal cut, it was observed that oxygen and carbon predominate and, in smaller quantities, potassium, phosphorus, sodium, iron, and magnesium.

On the other hand, when comparing the color characteristics of the epicardium of two species (Mapiría and Yucao), the main differences observed in color coordinates were in chroma and hue angle (Table 1). The samples of the Yucao variety indicate a bright red color, and the samples of the Mapiría variety are a bright and clear yellowish color. Greater brightness and vividness of color were detected in the Mapiría samples (Figure 4).

As for its sensory characteristics, it is considered an aromatic pseudofruit, sweet, refreshing, and fleshy, with an acid, astringent, wine-like, and almond flavor. However, this may change depending on the variety; some cashew apples can have a fruity aroma and be less acidic and fibrous or have less fleshy pulp, softer skin, and sweeter and exotic flavor [22,23,24].

Cashew is an undervalued and underutilized pseudofruit due to the lack of appreciation of its nutritional content by the agrifood industry and other sectors. It is acidic, with a pH between 3.5 and 5.0, which varies depending on the cultivar [3]. Its average crude fiber content is 3.5%, which provides a high amount of protein compared to other fruits [21]. Its content high in water (above 80%) and soluble solids (around 14 °Brix) are key factors. Among the predominant soluble sugars, glucose and fructose stand out with 5350 and 5170 mg/100 g f.w., respectively [25]. According to [13], fresh cashew fruit from Brazil has an 84.5% to 90.4% *w*/*w* moisture content, which matches with that described (85.6% *w*/*w*) by [25].

The values obtained for soluble solids show similarities with those presented by authors such as [21,26,27,28], among others; however, it is important to highlight the high content of soluble solids in these two varieties, which is similar to the values obtained by [22], who evaluated the chemical characteristics of three clones from Brazil, with similar and superior results to those of other authors.

Regarding the percentage of juice extracted (mean ± SD) from cashew fruit varieties in Colombia, Yucao stands out (88.7 ± 6% *w*/*w*; *n* = 10; own data) above others, including, for example, Mapiria (84.2 ± 2.4% *w*/*w*; *n* = 20; own data).

The pseudofruit is considered highly perishable due to its high water activity (Table 2), which causes waste due to the rapid growth of microorganisms. Another aspect to highlight is its low acceptability rate due to its astringency and sour or pungent taste, caused by the high tannin content and the presence of an unknown oily substance in the pericarp [3,29]. Different techniques for reducing tannins, such as clarification, microfiltration, fermentation, or heat treatments, have been proposed [30].

Regarding sensory acceptability, ref. [31] evaluated the sensory characteristics of cashew apple juice subjected to different heat treatments during a period of storage with microbiological control established. The authors of [31] used a 9-point hedonic scale applied to five different attributes and a panel of 30 untrained consumers. The juice was generally accepted due to the use of clarifying agents that reduce the tannin content and astringency.

On the other hand, ref. [32] elaborated a cashew apple jam, which was subsequently evaluated for its sensory acceptability with a panel of 28 people of both sexes and all ages. They evaluated nine attributes using a 10-point hedonic scale of 1 to 10 (liked extremely and disliked extremely). The study identified that the attributes with the highest score (7–9 points) were color, consistency, and sweetness. Medium scores (5–5.5 points) were obtained by color and general acceptability, and the lowest scores (2–3 points) were detected by the acid test, astringency, and aftertaste.

## 4. Processing Technologies: Cashew Uses and Applications

The processing of fruits and vegetables involves the reduction of the microbiological load of the food to increase its shelf life. Different fruit processing techniques, both thermal and nonthermal, have been used, although not all have been tested on cashews [33]. Recent treatments include ultraviolet radiation, ozone, high hydrostatic pressure, ultrasound, electrical pulses, freeze-drying, vacuum dehydration, enzyme-assisted treatments, fermentation, and others [34]. However, bioactive compounds and their preservation in plant matrices are affected during these processes, as is explained in the following section.

Cashew processing technologies (thermal and nonthermal) have been recently reviewed. The pseudofruit conveniently chopped after separation from the nut is subjected to dehydration processes by different procedures (osmotic dehydration, spray drying, freeze-drying) [35,36,37]. Other alternatives to maintain its quality consist of freezing [17] or rapid extraction of the juice by pressing and heating the bagasse [38]. In some cases, pulp cooking treatments (baking, boiling, steaming), frying, and even microwaving [39,40] are being tested (Table 3 and Table 4).

A process layout for the conceptual design of the valorization of cashews for energy, biofuels, biomaterials, and chemicals has been recently published [11,41]. The pseudofruit is used for juice extraction (usually containing between 65–85% juice), obtaining two types of juice: pulp and clarified juice. From these and with the addition of sugar, citric acid, and other preservatives, other products such as the following can be obtained: concentrated juices, syrups, jams, juices, and ready-to-serve beverages such as soft drinks, and others such as pickles, sweet and sour conserves, and fermented products, such as wine with 6% to 12% alcohol, ethanol, vinegar, and probiotic drinks [2].

Cashew apple juice can be fermented or transformed enzymatically to produce lactic acid, dextran, and oligosaccharides [42,43]. The pseudofruit can also be used as a feedstock to produce bioethanol in the form of fuel [3,21,24,44,45]. It can also be used as a sweetening agent, and components such as xylitol can be extracted from it. Moreover, the cashew apple allows the microbiological production of organic acids (lactic acid, acetic acid, oxalic acid); the isolation of bioactive metabolites and volatile compounds; the production of enzymes (dextransaccharase and tannase, among others); the production of biosurfactants; and the synthesis of nanoparticles, prebiotics, probiotics, and provitamins [11]. Lastly, the production of cellulose and lignin films using the pseudofruit has also been contemplated [29]. The kernel (nut) is used to produce oil, flour, butter, and Cashe Nut Shell Liquid (CNSL) [2].

After juice extraction, about 30% to 40% remains as bagasse, and only 10% to 20% of it is used in the industry [3]. According to chemical analysis by [46], bagasse contains about 58% moisture, 1.07% ash, 32% volatile compounds, 7.25% reducing sugars, 4.28% starch, and 14.2% cellulose. In dry form, bagasse is rich in fiber and is used to make fiber-rich cookies [3]. Bagasse has also been studied for the elaboration of high-fiber, low-fat patties and for protease extraction to be used as a meat tenderizer [47,48].

## 5. Effects of Preservation and Processing on Pseudofruit Nutrients and Bioactive Compounds

Bioactive compounds are of great interest in the food industry due to their antioxidant and antimicrobial properties and their possible contribution to the prevention and treatment of diseases such as obesity, type 2 diabetes, hypertension, and metabolic syndrome [49]. This is because some phenolic compounds can inhibit hyperglycemic enzymes such as α-amylase [49]. However, bioactive compounds are susceptible to oxidation reactions from storage to processing. Therefore, losses have been evidenced and attributed to the action of specific enzymes such as peroxidase and polyphenol oxidase, which participate in the oxidation of phenolic compounds and, therefore, may decrease the antioxidant properties. As for ascorbic acid, it is a very unstable antioxidant because its rate decreases due to light, temperature variations, and even interaction with oxygen [26]. Some studies suggest phenols may contribute significantly to antioxidant activity [50].

Macronutrients are more stable to different heat treatments, but micronutrients and bioactive compounds are sensitive to different heat treatments, where leaching and degradation processes occur, among others [40,51].

Some compounds are sensitive to different types of transformation, and nutrients are lost during the processes, even without applying heat. A study conducted by [52] considered the processing of cashew pseudofruit to obtain by-products. The authors evaluated cashews’ antioxidant properties and total phenol content and demonstrated their higher content in the unprocessed fruit and its juice (Table 3).

**Table 3:** Antioxidant properties and phenols of cashew by-products. Reproduced with permission from [52].

Product	FRAP R^2^:0.9991 (mMol Trolox· g^−1^)	ABTS R^2^:0.9955		DPPH R^2^:0.9865		Total Phenols (mg GAE 100 g^−1^)
		(mMol Trolox g^−1^)	% Captation	(mMol Trolox g^−1^)	% Captation	
Pseudo fruit	790.8 ± 10.7	615.3 ± 8.4	37.1 ± 0.4	388.5 ± 7.2	70.4 ± 1	37.0 ± 0.7
Fruit juice	415.7 ± 4.7	318.1 ± 8.4	40.3 ± 0.8	141.2 ± 0.2	56.7 ± 0.1	14.8 ± 0.9
Fruit juice + Aloe vera	456.4 ± 1.1	245.2 ± 7.3	32.7 ± 0.9	155.5 ± 1.4	63.7 ± 0.5	25.3 ± 0.1
Pseudo fruit flour	501.3 ± 7.1	872.3 ± 6.3	23.9 ± 1.6	71.5 ± 1.8	15.5 ± 0.1	26.9 ± 2.3
Dip Pulp fruit + pepper	341.4 ± 1.8	516.1 ± 3.9	32.4 ± 2.2	118.9 ± 7.5	29.1 ± 1	16.4 ± 0.1
Plain dip	485.7 ± 11.1	396.5 ± 4.8	22.5 ± 0.1	100.6 ± 7.3	24.2 ± 1	19.4 ± 0.9
Dip + red fruit	257.6 ± 8.7	280.4 ± 2.1	18.2 ± 41.2	87.7 ± 6.2	23.7 ± 0.9	19.0 ± 0.7
Dip + pineapple	371.8 ± 11.7	407.8 ± 3.8	22.2 ± 0.7	119.7 ± 5.9	26.1 ± 0.8	21.9 ± 0.1

Accordingly to [40], evaluated the impact of different cooking methods on the fiber and on other parameters of the pseudofruit, such as ascorbic acid, carotenoids, and total phenols. Significant differences between cooking methods were found due to the susceptibility of these compounds when exposed to different temperatures, the environment (oxygen), and the transfer of nutrients that occurs from the food to the cooking medium (leaching; Table 4). Cooking times, the compound’s solubility in the cooking medium, and the exposure temperature play an essential role in altering the cell walls and releasing these compounds.

**Table 4:** Cashew apple fiber’s bioactive compounds and the impact of different cooking methods on them. Data reproduced with Creative Commons CC-BY-NC-ND license from reference [40].

Fibre	Cooking Methods	Bioactive Compound or Group				
		Ascorbic Acid (mg/100 g)	Total Carotenoids (mg/100 g)	Total Phenols (mg GAE/100 g)	Total Antioxidant Activity (ABTS) μM Trolox g^−1^	Total Antioxidant Activity (DPPH) μM Trolox g^−1^
Artisanal	Before Cooking (Raw)	901.2 ± 74.88 ^a^	5.24 ± 0,37 ^c^	97.44 ± 6.65 ^a^	49.76 ± 18.96 ^d^	109.76 ± 13.66 ^a^
	Boiled	149.4 ± 18.0 ^d^	5.04 ± 0.50 ^c^	66.68 ± 5.91 ^c^	28.54 ± 12.91 ^e^	31.62 ± 1.89 ^f^
	Steamed	260.0 ± 15.7 ^c^	5.94 ± 0.14 ^c^	91.67 ± 0.67 ^ab^	81.59 ± 16.72 ^b^	78.82 ± 4.32 ^b^
	Frying	319.0 ± 11.7 ^b^	7.17 ± 1.16 ^bc^	43.45 ± 7.26 ^de^	104.95 ± 1.73 ^b^	71.42 ± 7.14 ^bc^
	Combined Oven	242.2 ± 11.4 ^c^	12.14 ± 1. 14 ^a^	95.69 ± 4.35 ^a^	74.66 ± 3.46 ^c^	61.82 ± 0.33 ^cd^
Industrialized	Before Cooking (Raw)	20.3 ± 0.02 ^d^	5.27 ± 0.04 ^c^	49.80 ± 0.22 ^d^	32.68 ± 7.05 ^e^	31.92 ± 2.31 ^f^
	Boiled	13.7 ± 1.0 ^e^	5.69 ± 0.85 ^c^	29.44 ± 3.20 ^e^	30.49 ± 4.50 ^e^	37.35 ± 2.94 ^f^
	Steamed	33.1 ± 3.7 ^d^	5.29 ± 0.20 ^c^	42.85 ± 4.43 ^de^	142.25 ± 6.50 ^a^	45.62 ± 3.99 ^ef^
	frying	27.5 ± 15.4 ^d^	10.18 ± 1.68 ^ab^	74.66 ± 7.05 ^bc^	83.85 ± 2.68 ^bc^	100.29 ± 1.67 ^a^
	Combined Oven	22.8 ± 5.6 ^d^	6.19 ± 0.81 ^c^	47.69 ± 1.58 ^d^	102.24 ± 7.62 ^bc^	51.11 ± 1.79 ^de^

Note: Data represents means ± SD on a dry basis. Means with a different superscript letter in the column are significantly different at *p* ≤ 0.05.

According to [51], ascorbic acid, in addition to being susceptible to oxygen or light, is also susceptible to processing at different temperatures (pasteurization, dehydration, blanching, cooking, sterilization, freezing). In short treatments such as preparing juice, the loss of ascorbic acid can go from 10% to 20%. In other cases, when there are no preventive steps, ascorbic acid loss can reach up to 100%.

On the other hand, ref. [39] evaluated the behavior of bioactive compounds, including the antioxidant activity in the pseudofruit’s fiber, by applying ultrasound at different intensity levels and using encapsulants such as maltodextrin and gum arabic. The authors concluded the application of ultrasound offers a good source of antioxidants compared to conventional methods.

To conclude, ref. [53] evaluated the behavior of ascorbic acid and carotenoids using two pasteurization methods (80 °C for 15 min and 94 °C for 29 min) and found that they do not have a significant impact on the processed juice. Moreover, ascorbic acid concentration and carotenoid concentration increased from 50% to 83%.

According to [54], the behavior of bioactive compounds in pasteurized orange juices (70–95 °C for 15–30 s) when adding maltodextrin resulted in favorable results: the ascorbic acid and vitamin C content of the samples with added maltodextrin was significantly higher than the control samples. Furthermore, maltodextrin had a protective effect on the total phenols rate, and the antioxidant activity was higher. In another pasteurization study conducted by [55], higher retention of phenolic compounds was achieved when processing the pseudofruit at 95 °C for 1 min.

On the other hand, a study conducted on cashew apple fibrousness using ultrasound showed higher retention of total phenolic compounds, with slightly higher vitamin C content and higher antioxidant properties [39]. Other sources mention that the use of ultrasound breaks cellular matrices and promotes the release of trapped molecules such as vitamins, antioxidants, polyphenols, and flavonoids in juice products, making them more bioavailable and bioaccessible [56].

Regarding the two types of pasteurization, a study carried out on pure kiwifruit using conventional pasteurization and microwave pasteurization showed that there was an impact on the carotenoid content in a similar way in both processes [57]. Nevertheless, the study mentions that the microwave process preserved to a greater extent the content of bioactive compounds, antioxidant activity, and chlorophyll content, generating a product with a color more similar to that of fresh fruit [57].

In the dehydration process, vitamin C decreases; the higher the temperature and the longer the time, the more the vitamin C decreases. On the other hand, the rate of carotenoids in dehydrated products can be higher due to the concentration effect. Moreover, a transformation process can occur due to the heat effect, and the total phenolic compounds increase, possibly due to the concentration effect [58]. However, osmotic dehydration using sucrose has been considered an alternative that preserves some sensory characteristics and allows for better texture and higher vitamin C rates than dehydrating with corn syrup [35]. In addition, ref. [37] states that the process mentioned previously reduces the destruction of flavonoids and increases the concentration of phenolic compounds, carotenoids, and antioxidant properties.

Lastly, when using a spray drying method, the wall material plays an essential role in the encapsulation efficiency of total phenols and other compounds [36].

As previously mentioned, for the thermal treatments, the frying process serves to a greater extent the content of bioactive compounds, probably because of the very high temperatures for a very short period. The high content of the fresh product can be evidenced, but the difficulty is its short shelf life. Therefore, thermal and nonthermal treatments allow shelf-life extension. The use of nonthermal treatments and the spray drying process preserves the compounds of most significant interest to a greater extent [36].

The treatments used show favorable results when keeping the concentrations of some compounds, such as frying, and some compounds in the boiled methods. However, the means used to apply these types of processing (oil/water), the temperatures used, and the time of processing can affect the concentration of the compounds. For example, β-carotenes are more soluble in oil, and ascorbic acid is more soluble in water. Also, an increased relative concentration due to dehydration processes (water loss of the food matrix) occurs.

## 6. Composition in Bioactive Compounds and Potential Uses

Oxygen, abundant and essential for the development of life, also has a negative effect on the body’s aerobic metabolism since it produces reactive oxygen species (ROS), free radicals, and non-radicals. If the balance between the overproduction of ROS and the body’s antioxidant defense mechanism is positive, oxidative stress occurs, along with the breakdown of cellular function and damage due to oxidative stress [59,60].

In recent decades, trends have shown essential changes in the diet where the consumption of compounds in food promotes health and reduces the risk of disease [61]. In this sense, the cashew pseudofruit, besides being considered a source of nutrients, is also considered a source of bioactive compounds, highlighting the content of carotenoids, anthocyanins, total phenolic compounds, minerals, flavonoids, ascorbic acid and volatile organic compounds (Table 5 and Table 6) [2,21,62]. The analysis of cashew apples showed high values for three parameters (55.8 mg/100 g of vitamin C, 603 mg/100 g of TPC and 924 μg/100 g of carotenoids) [63]. Therefore, due to its composition, this pseudofruit has the potential for elaborating functional foods and dietary supplements due to its high nutritional and nutraceutical value.

According to [31] cashew apples contain 5 times more vitamin C than orange juice and 10 times more than pineapple juice.

Numerous antioxidants (flavonoids, carotenoids, and anthocyanins) can be extracted from the pseudofruit residue and could potentially be used in the meat, dairy, and bakery industries [3]. The use of the cashew pseudofruit fiber for human consumption opens a new perspective as a natural source of phenolic compounds and antioxidant activity [62].

In the cashew pseudofruit extract, ref. [38] identified catechins, quercetins, myricetins, and esters of ethyl hydroxybutanoic acid, among others, which are protective substances against free radical damage (high antioxidant properties), and some of them have anti-inflammatory properties.

The content of flavonoids (myricetin, quercetin, and kaempferol) and flavonols (luteolin and apigenin) in Brazilian fruits (mango, papaya, acerola, apple, cashew apple, guava, orange, jaboticaba-or jaboticaba-, pitanga, strawberry) was evaluated by HPLC [71]. The authors reported myricetin and kaempferol in cashew apples, but they were undetectable in mango and papaya. They also highlighted the cashew apple, pitanga, acerola, and apple as the best sources of flavonols.

Bioactive compounds are considered compounds from plants, which have developed them as a defense mechanism against ultraviolet radiation, pathogens, and herbivores [72]. Bioactive compounds are classified by their clinical, pharmacological, and toxicological effects. The pseudofruit is a source of carotenoids, ascorbic acid, anacardic acid, tannins, gallic acid, phenolic compounds, and flavonoids such as gallic acid, caffeic acid, coumaric acid, ferulic acid, quercetin, myricetin, among others. The flavonoids usually occur in the form of glucosides [70,72,73,74].

From a biological point of view, the compounds previously discussed offer health benefits, and many of these benefits are attributed to their antioxidative capacity, especially flavonoids. In a study by [75], polyphenols such as epicatechin inhibited the oxidation of LDL (low-density lipoprotein) 2 h after consumption of cocoa, which has a considerable amount of the compound in question. On the other hand, flavonoids have biological and pharmacological importance because they have anticancer, anti-inflammatory, antiviral, antiallergic, antioxidant, and chelating activities, reducing deaths from ischemic heart disease, among others. Quercetin and tannins inhibit the neuronal constitution of nitric oxide synthase (NOS) [71,73].

## 7. Bioaccessibility, Bioavailability, and Health Effects of Pseudofruit Bioactive Compounds

It is important to consider the bioaccessibility of phenolic compounds. However, the amounts that are not absorbed in the intestine undergo transformation and degradation processes by the colon microflora, where high molecular weight phenolic compounds are degraded to low molecular weight compounds that may be biologically more active, better absorbed, and could even possess prebiotic activities and stimulate the growth of beneficial microflora such as bifidobacteria and lactobacillus, among others [76].

De Lima et al evaluated the compounds in the juice and fiber of the pseudofruit (minerals: copper, iron, zinc, ascorbic acid, and total polyphenols and antioxidant activity) that become available during gastrointestinal digestion for intestinal absorption, which is expressed as bioaccessibility. It was evidenced that in the fiber, the amount of minerals, ascorbic acid, and total polyphenols, as well as antioxidant activity, decrease during gastrointestinal digestion. On the other hand, bioaccessibility is higher after digestion of the juice for the minerals copper, iron, and zinc, with percentages of 15%, 11.5%, and 3.7%, respectively. These data more than doubles (except zinc) compared to the bioaccessibility obtained from the fiber, with percentages of only 4%, 1.2%, and 2.2% for copper, iron, and zinc, respectively [62].

As for ascorbic acid, total polyphenols extracted, and antioxidant activity, according to [62], its behavior was similar being the contents more than doubled in juice in comparison to fiber, with bioaccessibility values of 26% ascorbic acid, 39% polyphenols, and 27% antioxidant activity in the juice, while for the fiber, ascorbic acid was not detected. As for polyphenols and antioxidant activity, values of 16.6% and 10.2%, respectively, were found. The bioaccessibility values in mg·L^−1^ for each can be calculated according to the data recorded in Table 4, identified with B and C [62].

Therefore, the bioavailability of nutrients depends on bioaccessibility (i.e., the higher the amount of nutrients released from the food during the gastrointestinal process, the higher the amount of nutrients available to act on the human organism).

**Figure 6:**
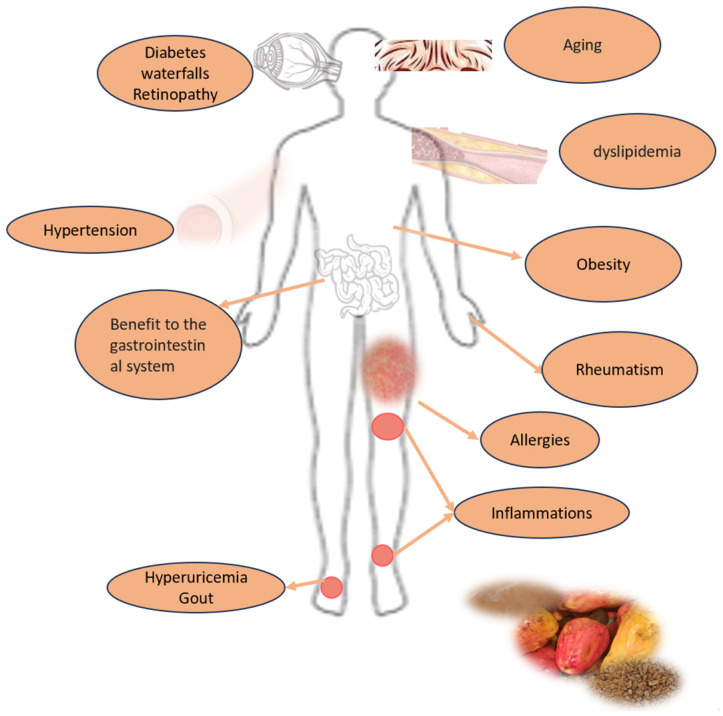
Potential human health benefits of *Anacardium occidentale* pseudofruit consumption.

Some studies and patents show the content of active compounds in cashew apples and their positive impact on health, addressing different chronic non-communicable diseases and others. Due to its therapeutic components (phenolic compounds) and its antioxidant activity, the pseudofruit could be considered an agent for treating obesity, an anti-inflammatory agent, and an antioxidant food with antimicrobial, antidiabetic, and other properties (Figure 6; Table 7) [9,43,44,49].

The richness of phenolic contents in the cashew pseudofruit pulp puree used for a patent by [9] allowed the authors to identify intense antioxidant activity and its usefulness as an inhibitor of lipid autooxidation and as an active ingredient of a whitening agent (Table 7). In addition, these phenolic compounds may be antiaging agents because they prevent elastin degeneration in the skin and the like and help reduce wrinkles and other diseases [9].

### 7.1. Potential Health Benefits of the Pseudofruit and Its Different Extracts

Consuming unprocessed pseudofruit extract has a protective effect on high-fat diets, intestinal health, and lipid metabolism. Therefore, it could be considered a dietary supplement or an ingredient for elaborating edible products [77].

### 7.2. Puree of Freeze-Dried Pseudofruit Peel, Pulp, and Seeds

In [44], the pseudofruit puree was used to evaluate in vitro changes in the microflora under simulated gastrointestinal digestion. It showed that its fermentation decreased the pathogenic enterobacteria group as well as the pH and exerted a prebiotic effect on the intestinal microflora due to its fiber content (total 35% and insoluble 27.7%). An increase in microorganisms associated with human health was evidenced, in addition to an increase in the production of acetic, butyric, and propionic acid during colonic fermentation. Its probiotic potential indicates that it can be used to formulate supplements.

These purees improved intestinal health and lipid metabolism in rats with diet-induced dyslipidemia [27]. The main observed effect was the reduction of weight gain and controlled, lower levels of TG and total and LDL cholesterol and an increase in HDL (protective against atherosclerosis). In addition, the puree compounds showed signs of preventive action in lipid metabolism, fecal pH, liver fat accumulation, colonic epithelial cell integrity, and liver cell structure. Therefore, the cashew apple may be a potential dietary supplement or food ingredient.

**Table 7:** Potential beneficial effects considering different tests (inhibition tests) and results obtained of cashew pseudofruit (*Anacardium occidentale*) pulp puree according to [9].

Test Performed	Result Obtained	Effect and Potential Practical Application
Superoxide and hydroxyl radical scavenging activity	Free radical scavenging.	Not registered.
Antioxidant activity of linoleic acid-β-carotene	Suppresses linoleic acid autooxidation.	Lipid autooxidation.
Tyrosinase inhibitory activity	Inhibition.	Active ingredient of a bleaching agent.
Elastase inhibitory activity	Inhibition.	Prevention of elastin degeneration in the skin and the like. Suppression of skin aging (wrinkles). Prophylactic or therapeutic agent (e.g., rheumatism).
Collagenase inhibitory activity	Inhibition.	Antiaging agent.
Hyaluronidase inhibitory activity	Inhibition.	Prevention or treatment of diseases caused by hyaluronidase activity (inflammation and allergy).
Collagen aging	Suppression of the formation of collagen cross-linking.	Antiaging agent.
Inhibitory activity of α-amylase and α-glucosidase	Inhibition of both enzymes.	Delays the glucose spike that occurs after a meal. Potential active ingredient of a prophylactic or therapeutic agent (conditions or diseases related to both activities; inhibitor of increased blood glucose level, diabetes, etc.).
Inhibition of production of Advanced Glycation Endproducts (AGE)	Inhibition of protein glycation end-product generation.	It can be used as an active ingredient in a prophylactic or therapeutic agent against diseases involving AGEs, such as diabetic complications.
Lipase inhibitory activity	Inhibition.	Treatments against obesity and hyperlipidemia, suppressing lipid absorption after ingestion. Suppression of lipid degradation and its odors that are produced by lipase and microorganisms.
Angiotensin-converting enzyme (ACE) inhibition	Inhibition.	Active ingredient of a prophylactic or therapeutic agent against a condition or disease (such as hypertension) by inhibiting angiotensin-converting enzyme activity).
Urease inhibition	Inhibition.	Preventive or therapeutic effects for diseases involving Helicobacter pylori.
Xanthine oxidase (XOD) inhibitory activity	Inhibition.	Active ingredient of a preventive or therapeutic agent against a condition or disease (hyperuricemia, ventilation, gout, etc.).

### 7.3. Pseudofruit Residue (Obtained from Processing after Extracting Juice)

In an in vitro study [49] on the bioaccessibility of polyphenols, flavonoids, and antioxidant properties, in addition to evaluating α-amylase inhibition, the authors demonstrated that hydrolyzable tannins play an important role in antioxidant activities and as a free radical scavenger. Their results showed a high α-amylase inhibitory activity during in vitro digestion and high stability of phenolic compounds, suggesting that these components of the pseudofruit residue may be useful for pharmacological applications, for the development of functional foods, and for the therapeutic treatment of diabetes.

### 7.4. Pseudofruit Ethanolic Extract

Mice treated with this extract and under an atherogenic diet showed a reduction in weight gain compared to the control. This extract could inhibit α-amylase, pancreatic lipase, and lipid absorption [69]. The ethanolic extract had an impact on the lipid profile markers, liver function (decreasing enzymatic markers), and lipid accumulation of the mice (reduced atherosclerotic wall narrowing of the arterial lumen).

Anacardic acid has shown excellent potential to inhibit proliferation and induce apoptosis of prostate cancer cells in prostate cancer cell lines [78].

### 7.5. Hydroalcoholic Pseudofruit Extract

A study conducted by supplying hydroalcoholic extract of the pseudofruit to mice evaluated the preventive and curative effects on body weight gain, fat storage, hyperuricemia, hyperinsulinemia, and insulin resistance. The consumption of 20 mg/kg of pseudofruit extract prevents body weight gain and significantly reduces blood glucose levels. It also prevents accelerated increase of insulin levels and reduces resistance to it [79].

### 7.6. Pseudofruit Juice or Pseudofruit Proanthocyanidin Extract

The proanthocyanidin extract from pseudofruit juice exhibits high inhibition of α-amylase (IC50 of 1.1 μg/mL) and lipase (IC50 of 0.5 μg/mL), as well as low antimicrobial activity [80]. Therefore, this extract is a potential active or therapeutic ingredient to prevent diabetes, obesity, hyperlipidemia, acne, and lipid spoilage.

In mice, the effects of pseudofruit juice depend on the degree of maturity of the pseudofruit [43]. Unripe fruit juice presented 65% higher anti-inflammatory activity and improved the healing process by 88.3% compared to ripe fruit juice. In addition, unripe fruit juice improved immune defense mechanisms and balanced reactive oxygen species and antioxidants.

The information previously described has been condensed in the Supplementary Table S1.

## 8. Conclusions

Cashew pseudofruit has different characteristics from its composition, highlighting in the latest research its content of bioactive compounds such as ascorbic acid, carotenoids, proanthocyanidins, and some phenols related to possible benefits. However, this is a highly discarded product worldwide that has attracted the attention of farmers, from pre-harvest techniques to postharvest techniques, and in the food industry where research has been found that offers alternatives of thermal and nonthermal treatments and the behavior of these bioactive compounds in such a way that they provide an alternative to maintain their quality and increase their shelf life. Studies show it is a product with high potential and possible health benefits for the consumer. It offers an excellent opportunity to develop different functional foods and applications in the food industry.

By offering new preservation alternatives, the producer and industry can focus their attention on developing new products, of which there is currently limited, and even considering their potential health benefit, could be included or used for the elaboration of functional foods. In-vivo studies, especially in mice, showed satisfactory and promising results of the bioactivity of some of the pseudofruit extracts. New studies on humans are necessary to confirm such evidence and get new products that are useful for developing functional foods or other uses.

## Figures and Tables

**Table 1:** Range of epidermis color coordinates for two cashew pseudofruit varieties of different coloration (yellow or red) from the city of Puerto Carreño (Colombia). The HunterLab ColorQuest XE (Lanzetta Rengifo Bogotá, Colombia) was calibrated with the D65 observer, and the vision was 0° (own data).

Variety	Lightness (L*)	Chroma (C*)	Hue Angle (°)
Mapiría	57.07–67.63	64.04–89.85	75.32–82.96
Yucao	31.22–53.28	51.24–76.35	36.23–65.23

**Table 2:** Chemical and compositional characteristics of cashew pseudofruit varieties Mapiría and Yucao from the Colombian Orinoco region (own data based on fresh weight with the exception of fibers based on dry weight).

Parameter	Mapiría	Yucao
pH	4.70	4.27
Aw (water activity)	0.96	0.96
Total soluble solids (°Brix)	13.4	14.4
Acidity (g/100 mL juice of malic acid)	0.21	0.20
Protein (g/100 g)	0.47	0.55
Moisture (g/100 g)	84.1	84.2
Ash (g/100 g)	0.02	0.02
Lipid (g/100 g)	1.79	1.74
Crude fiber (g/100 g)	0.9	0.9
Hemicellulose (g/100 g d.w.)	0.9	0.7
Cellulose (g/100 g d.w.)	1.4	1.3
Lignin (g/100 g d.w.)	0.2	0.2
Glucose (g/100 g)	0.51	0.45
Fructose (g/100 g)	0.49	0.44
Myo inositol (mg/g)	0.04	0.04
D-pinitol (mg/g)	0.01	0.01
Calcium (mg Ca/100 g)	5.4	6.2
Iron (mg Fe/100 g)	2.2	2.4
Sodium (mg Na/100 g)	2.6	2.7
Total phosphorus (mg P/kg)	3.1	2.6

Note. Average value obtained from the analyses performed on the whole pseudofruit.

**Table 5:** Content of other nutrients, according to different authors and basis of measurement (dry, wet, extract, or juice) in the cashew pseudofruit.

Nutrient Compound	Unit	Average	References
Potassium	mg/g	4.92 ^a^	[28]
Copper	mg/g	0.004 ^d^	[63]
Magnesium	mg/g	1.03 ^a^	[28]
Manganese	mg/g	0.02 ^a^	[28]
Zinc	mg/g	0.01 ^a^	[28]
Ascorbic Acid	mg/100 g	30.5 ^a^	[28]
		218.9 ^d^	[21]
		198.5 ^e^	[22]
		190 ^d^	[64]
		243.8 ^d^	Own Data *
	mg/g	0.49 ^e^	[61]
	mg/g	2.561 ^f^	[26]
	mg/g	2.771 ^f^	[65]
	mg/g	2.52 ^a^	[25]
Beta-Carotene	μg/g	4.542 ^f^	[66]
	μg/g	7.60 ^a^	[25]
	mg/100 g	0.69 ^f^	[67]

Note: The conventions used in the table correspond to specific conditions of the analyzed pseudofruit: ^a^ Quantity in the edible part of the fruit; ^d^ Quantity in whole fruit; ^e^ Average quantity of cashew stalks; ^f^ Quantity in the dry basis of pulp. * Determined by HPLC chromatography.

**Table 6:** Content of bioactive compounds and antioxidant properties according to different authors and basis of measurement (dry, wet, extract, or juice) in the cashew pseudofruit. GAE, gallic acid equivalent. DPPH (2,2-diphenyl-1-picrylhydrazyl).

Nutrient Compound	Unit	Average	References
Total Phenols	mg GAE/100 g	13.20 ^a^	[28]
		365.303 ^d^	[21]
		326.80 ^e^	[22]
		802.2 ^f^	[27]
		118 ^f^	[65]
		830 ^f^	[65]
		61.1 ^f^	[66]
		338.6 ^b^	[68]
		566.1 ^c^	[69]
		5286.5 ^f^	[67]
	mg GAE/kg	122.8 ^f^	[68]
	mg GAE/g	209.6 ^f^	[69]
Anacardic Acid 15:1	mg anacardic acid/g	2.11 ^f^	Own Data *
Anacardic Acid 15:2	mg anacardic acid/g	1.50 ^f^	Own Data *
Anacardic Acid 15:3	mg anacardic acid/g	1.23 ^f^	Own Data *
Anthocyanins	mg/100 g	2.5 ^a^	[28]
		7.6 ^f^	[67]
		9.5 ^d^	[65]
Antioxidant Activity	μM trolox g^−1^	18.1 ^b^	[67]
	μM trolox g^−1^	51.1 ^c^	[67]
	μM trolox g^−1^	79.4 ^f^	[65]
	DPPH reduction %	94 ^f^	[68]
	DPPH reduction %	57.5 ^f^	[68]
	DPPH reduction %	68.1 ^f^	[69]
Flavonoids	mg/kg	122.8 ^f^	[68]
	mg/100 g	63.8 ^d^	[65]
	mg/100 g	301 ^f^	[27]
	mg/g	249.6 ^f^	[69]
Total Glycosylated Flavonoids	mg/g	0.2847 ^f^	[70]
Total Quercetin Glycosides	mg/g	0.1139 ^f^	[70]
Total Myricetin Glycosides	mg/g	0.1511 ^f^	[70]
Condensed Tannins	mg/100 g of catequine	3.04 ^g^	[49]
Hydrolyzable Tannins	mg/100 g of tannic acid equivalent	736.35 ^g^	[49]

Note: The conventions used in the table correspond to specific conditions of the pseudofruit analyzed: ^a^ Quantity in the edible part of the fruit; ^b^ Quantity in fruit juice; ^c^ Quantity in fruit fibrousness; ^d^ Quantity in whole fruit; ^e^ Average Quantity of 3 clones of cashew peduncles; ^f^ Quantity in the dry basis of pulp; ^g^ Pseudofruit waste, * They were performed by UPLC-QTOF-MS method, using an RT retention time directed MS/MS, quantifying with anacardic acid standard 15:3 from sigma Aldrich and quantifying anacardic acids 15:1, 15:2 by equivalence.

## Data Availability

No new data were created or analyzed in this study. Data sharing is not applicable to this article.

## Supplementary Materials

The following supporting information can be downloaded at https://www.mdpi.com/article/10.3390/foods13152357/s1, Table S1: Consolidated in-vitro and in-vivo studies were carried out with cashew apple pseudofruit (*Anacardium occidentale*).
