# Supplementary material for: The Cashew Pseudofruit (Anacardium occidentale): Composition, Processing Effects on Bioactive Compounds and Potential Benefits for Human Health
Source: Foods. 2024 Jul 26;13(15):2357. doi: 10.3390/foods13152357 (PMC11311269; doi:10.3390/foods13152357)
Supplement: Supplementary file 1 [file foods-13-02357-s001.zip › foods-3101041-supplementary.pdf]

foods

Supplementary Information

The Cashew Pseudofruit (*Anacardium occidentale*): Composition, Processing Effects on Bioactive Compounds and Potential Benefits for Human Health

Carina Gutiérrez-Paz [1,2], María-Constanza Rodríguez-Moreno [2], María-Soledad Hernández-Gómez [1] and Juan Pablo Fernández-Trujillo [3,*]

[1] Instituto de Ciencia y Tecnología de Alimentos (ICTA), Universidad Nacional de Colombia, Carrera 30 Calle 45, Bogotá 111321, Colombia; cgutierrezp@unal.edu.co (C.G.-P.); maria.rodriguez@unicafam.edu.co (M.-C.R.-M.); mshernandez@unal.edu.co (M.-S.H.-G.)
[2] Centro de Pensamiento Turístico de Colombia, Escuela de Turismo y Gastronomía, Fundación Universitaria Cafam, Ak 68 #90-88, Bogotá 111211, Colombia
[3] Department of Agronomical Engineering, Technical University of Cartagena, Paseo Alfonso XIII, 48, ETSIA, 30203 Cartagena, Murcia, Spain
* Correspondence: juanp.fdez@upct.es

Supplementary Table S1. Consolidated in-vitro and in-vivo studies were carried out with cashew apple pseudofruit (*Anacardium occidentale*).

Test performed	Result obtained	Effect	References
	**In-vivo**		
**Experimental animal:** Swiss mice.<br>**Product supplied:** Ripe and unripe cashew apple.<br>**Assessment:**<br>antioxidant capacity,<br>anti-inflammatory activities<br>healing activities<br>**Methodology:**<br>The subjects were separated into groups.<br>-Ear edema was induced to evaluate the anti-inflammatory capacity.<br>**1.** The control group was treated with dexamethasone,<br>**2.** Two groups were pretreated with juice, one group for each type of juice,<br>**3.** The case of healing activity a wound excising skin of 1cm2 in the dorsal region.<br>**3.1** Two groups were pretreated daily by gavage with mature and unripe juice.<br>**3.2** The control group received water, and another group received nutritional supplement.	**Antioxidant capacity:**<br>The juice of ripe fruit had twice the antioxidant capacity and higher levels of total phenols, anthocyanins, yellow flavonoids, and vitamin C than that of unripe fruit, except for carotenoids and tannins.<br>**Anti-inflammatory activity**:<br>The group treated with unripe fruit juice presented better anti-inflammatory activity 66.5% with a similar response to the control group (dexamethasone 75%)<br>**healing activities**:<br>The group treated with the immature juice had less inflammation in the wound and thinner granulation tissue.<br>The groups treated with supplement and unripe fruit juice presented lower unhealed wounds at 14 days, with a wound contraction for those treated with the unripe juice of 86%	Unripe fruit juice: better anti-inflammatory properties and healing activities, probably attributed to its phytochemical components improving immunological defense mechanisms and balancing oxygen-reactive and antioxidant species, leading to a better healing process.<br>Ripe fruit juice: Low healing and inflammatory response to the pretreatment that increased the level of antioxidants, causing a redox imbalance.	[43]
**Experimental animal:** Adult male Wistar rats weighing 180-200 g<br>**Product supplied:** Fuit fresh<br>**Assessment:**<br>Inhibition of lipid metabolism and amelioration of obesity in rats.<br>**Methodology:**<br>**Model 1.** Lipid emulsion model by assessing postprandial plasma triglyceride levels.<br>**Model 2.** Obese rats induced by atherogenic diet.<br>An evaluation of the serum lipid profile was performed.<br>Histopathological studies of the carotid artery and liver were also performed.<br>The rats were divided into five groups.	**Model 1.**<br>The treatment with ethanol extracts of psudofruit at 200 and 400 mg/kg and a standard drug (atorvastatin) reduced the level of plasma triglycerides from the first hour onwards compared to the control group of obese rats, at 60 days, showed a reduction in body weight, and the index mass body (BMI).<br>**Model 2.** The administration of 400 mg/kg of ethanol extract from cashew apple showed a behavior similar to that of atorvastatin. It notably reduced TG, LDL, and VLDL cholesterol in plasma and increased HDL, similar to the effect	The ethanolic extract of pseudofruit can inhibit the absorption of dietary lipids.<br>The ethanolic extract of pseudofruit can be a potent nutritional supplement or adjunctive therapy for treating obesity, atherosclerosis, and other metabolic disorders.<br>They are considered to have a potent antiobesity effect	[69]

Test performed	Result obtained	Effect	References
**Group 1.** Control, 1% carboxymethylcellulose was administered. **Group2.** Received atherogenic diet and served as obesity control. **Group 3.** Received atherogenic diet and standard drug atorvastatin 10mg/kg. **Groups 4 and 5.** received an atherogenic diet with ethanol extract of cashew apple at 200mg/kg and 400 mg/kg. The animals were anesthetized on day 60, administered ketamine and xylazine, and finally sacrificed by cervical dislocation.	shown by the standard drug. It reduced the atherogenic index, a marker for cardiovascular diseases and cardioprotective. It improved liver function by decreasing enzymatic markers such as AST, ALT, and ALP, also increased SOD, GSH, and CAT levels, and reduced MDA levels, inhibiting lipid accumulation in the liver. It reduced the atherosclerotic narrowing of the arterial lumen.		
**Experimental animal:** mouse with diet-induced obesity. **Product supplied:** cashew apple extract hydroalcoholic extract. **Assessment:** to evaluate the preventive and curative effects of reducing body weight, fat storage, hyperglycemia, hyperinsulinemia, and insulin resistance. **Methodology:** **Experimental design 1**. In preventive design, mice were orally treated with Cashew Apple Extract at 200 mg/kg body weight from day one on a high-fat diet and for eight weeks afterward. **Experimental design 2.** In curative design, the animals were first maintained on a high-fat diet for four weeks and then treated with Cashew Apple Extract for four more weeks on the same regimen.	In the group treated with cashew apple extract for both experimental designs, a reduction in weight gain due to reduced fat storage in the liver, compared to the control group, also significantly reduced blood glucose levels, preventing the rapid increase in insulin levels and reduced insulin resistance.	The consumption in mice of 200mg/kg body weight of pseudofruit extract, in both preventive and curative designs, resulted in a significant reduction in body weight gain, fat storage, hyperglycemia, hyperinsulinemia, and insulin resistance. Therefore, it can be considered an attractive ingredient for managing obesity.	[79]
**Experimental animal:** female Wistar rats with diet-induced dyslipidemia **Product supplied:** industrial processing by-products of acerola, cashew, and guava fruits. The fruit was administrated (400 mg/kg body weight) via orogastric for 28 consecutive days. **Assessment:** effects of diet supplementation on the intestinal health and lipid metabolism	Weight loss was evidenced compared to the control group; it was more remarkable in acerola and cashew apple than guava, with the supply of the three fruits, lower levels of TG, total and LDL cholesterol, and an increase in HDL levels, considering it as a protector against atherosclerosis. The consumption of the pseudofruit effectively prevented lipid metabolism	They consider that the consumption of the three fruits has a protective effect against the damages caused by a dyslipidemic diet in the case of lipid metabolism and intestinal health. Therefore, it could be considered a dietary supplement or an ingredient in the elaboration of food products.	[27]

Test performed	Result obtained	Effect	References
**Methodology:** Rats were divided into five groups: **Group 1:** Healthy control, **Group 2:** Dyslipidaemia control **Group 3 to 5:** Dyslipidaemia experimental receiving acerola, cashew, or guava.	changes induced by a dyslipidemic diet. Pseudofruit consumption reduced weight gain, fecal pH, and fat accumulation in the liver, and preserved the integrity of colonic epithelial cells and liver cell structure.		
**Experimental focus:** Human prostate carcinoma cell line. **Assessment:** Evaluate anticancer effects of anacardic acid on cell apoptosis of prostatic cancer. **Methodology:** Cells were treated with 12.5, 25, and 50 μM of anacardic acid for 24, 48, and 72 h.	Anacardic acid inhibits proliferation and suppresses cancer cells, induces prostatic cancer cell apoptosis through autophagy of prostatic cancer cells, suppresses prostatic cancer cells	They point out that anacardic acid shows excellent potential and could be used as a new drug for the therapy of prostate cancer and that it could also have a positive effect on ovarian cancer.	[78]
	**In-vitro**		
**Assessment:** Changes in gastrointestinal microflora and pH during in vitro fermentation, assessment in human fecal samples **Evaluated sample:** Freeze-dried sample of peels, pulp, and seeds of the pseudofruit.	The sample presented a high total fiber content of 35%, with insoluble fiber standing out at 27.7%. In the digestion process, the availability of carbohydrates resistant to digestion and absorption in the small intestine was evidenced when the powdered sample was incorporated into colonic fermentation in vitro; relatively abundant positive changes were evidenced in the group of bacteria found as part of the human colonic microflora associated with prebiotic properties.	Increases the population of *Bifidobacterium* and *Lactobacillus/Enterococcus,* which are associated with human health, and decreases the count of enterobacterium C. histolyticum, which has been recognized as a pathogen, so it has a positive effect on the intestinal microflora. It decreases the pH and increases the production of acetic, butyric, and propionic acid during colonic fermentation, reinforcing its potential prebiotic effect that can be used in the formulation of supplements.	[44]
**Assessment:** Bioaccessibility of polyphenols, flavonoids, and antioxidant capacity simulated gastrointestinal digestion and pro-biotics fermentation by UFLC-DAD. Inhibition of α-amylase. **Evaluated sample:** fruit residues	The content of condensed tannins and hydrolyzable tannins identified in the pseudofruit (3.04mg/100g and 736.35 mg/100g respectively) play an essential role in the activities as an antioxidant and free radical scavenger, high inhibitory activity of α-amylase was evidenced (concentration of 0.1 $mg*mL^{-1}$ inhibited 57.83% and 83.99% of the enzyme in aqueous and ethanol extracts, respectively), and a high	Suggests that the pseudofruit is promising for the therapeutic treatment of diabetes because of its high inhibition of α-amylase. It can potentially be explored with functional food and pharmacological applications	[49]

Test performed	Result obtained	Effect	References
	antioxidant activity. During the in-vitro digestion, it was evidenced that the phenolic compounds presented high stability, which can be considered highly bioaccessible and potentially bioavailable.		
**Assessment:** Inhibitory capacity of α-amylase, lipase, and antibacterial activity. **Evaluated sample:** Extraction of proanthocyanidin from pseudofruit.	Proanthocyanidins are part of the flavonoid groups, especially a condensed tannin extracted from the pseudofruit, which was identified as having a solid α-amylase inhibitory activity with IC50 of 1.1 μg/ml, as for lipase inhibitory activity with IC50 of 0.5 μg/mL and antibacterial activity where at concentrations of 12.5 μg/ml there is no evidence of high effectiveness against propionibacterium acnes.	Use as an active or therapeutic ingredient for diabetes, obesity, hyperlipidemia, acne, and lipid spoilage.	[80]
